# Chronic Neuropsychological Sequelae in a Patient with Nontumorous Anti-NMDA-Receptor Encephalitis

**DOI:** 10.1155/2017/5675732

**Published:** 2017-04-19

**Authors:** Dong Y. Han, Lisa M. Koehl, Aarti Patel, Zhengqiu Zhou, Sarah Phillips, Siddharth Kapoor

**Affiliations:** ^1^Department of Neurology, University of Kentucky College of Medicine, Lexington, KY, USA; ^2^Department of Neurosurgery, University of Kentucky College of Medicine, Lexington, KY, USA; ^3^Department of Physical Medicine & Rehabilitation, University of Kentucky College of Medicine, Lexington, KY, USA; ^4^Department of Psychiatry, University of Kentucky College of Medicine, Lexington, KY, USA

## Abstract

Anti-N-methyl-D-aspartate receptor encephalitis is a neurological, autoimmune disorder tightly conceptualized only as recently as the mid-2000s. It presents itself in a combination of psychiatric, neurological, and autonomic features. We observe a unique case with probable earlier episode (prior to the mid-2000s conceptualization of the disease) and a later relapse, accompanying a comprehensive neuropsychological profile tracked after the relapse and subsequent improvement. Neurocognitive findings revealed residual frontal deficits with mood changes even in the state after plasmapheresis. This case is the first to describe posttreatment cognition in anti-NMDAR encephalitis after probable serial autoimmune episodes.

## 1. Introduction

Anti-N-methyl-D-aspartate receptor (anti-NMDAR) encephalitis is a neurological, autoimmune disorder that presents itself in a combination of psychiatric, neurological, and autonomic features. Typical representations include generally acute personality and behavioral changes, memory loss, hallucinations, dyskinesia, dystonia, and seizures. Significant portions of the patients, up to 80%, are women with a median age in the early twenties, and ovarian teratomas and related autoimmune processes are found in approximately half of the cases [1–5]. In this case report, we present a patient with two paroxysms of anti-NMDAR encephalitis accompanied by limited cognitive abilities, frontal deficits, and mood changes.

## 2. Case Presentation

The patient is a 33-year-old married woman. She managed the financial aspects and business ventures of the three companies that she and her husband own. Her social history was reported normal with no incidents of significant trauma or substance abuse. Developmental history is unremarkable, and she completed one and a half years of college. The family history showed bipolar disorder in one sibling, hypertension, diabetes, and a strong presence of depression.

Upon records review, documentation revealed that the patient possibly, or rather probably, experienced her first episode of autoimmune encephalitis at the age of 24, which occurred prior to the first conceptualization documented by Dalmau et al. [3]. This episode was paired with headaches, hallucinations, delusions, violent outbursts, seizures, and then a two-month long coma. During this time, hallucinations included seeing a television on the ceiling. She was hospitalized at an inpatient psychiatric facility, but details of her clinical diagnoses at the time remain unclear. She then recovered drastically after receiving a high dose of prednisone and only reported mild to moderate residual deficits. While no formal neuropsychological measures were given at the time per report, residual deficits included low-level math computation, slowed processing speed, and short-term memory deficits, enough to a level that required supervision over daily living activities. This continued to resolve for about 2 years until she was able to drive and care for her children independently. However, she remained agitated and irritable compared to her baseline.

The second episode occurred nine years later and was much more severe. It involved personality changes, perseveration in behaviors and speech, impulsive financial behaviors (e.g., spending over $10,000 on clothes in one week), paranoia, psychosis, and aggressive outbursts. She also had dressing apraxia, and her psychosis included visual hallucinations of monsters and demons, melting ceilings, delusions of her children being trapped under the bathroom tiles, and the belief that she had to free them using only her fingernails. She was under restraint during her inpatient stay for a week due to aggressive outbursts. She also developed status epilepticus 4 times, which were treated with conventional IV antiepileptic drugs.

Video EEG showed epileptic activity in the right frontoparietal region. The remainder of the workup was negative, including MRI, CT, PET, transvaginal US, and LP. MRIs of the brain and pelvis were both negative (pelvic MRI was initiated to rule out ovarian teratoma). CSF was unrevealing including T. pallidum, West Nile, IgG, albumin, cell ct/protein/glucose, cultures, cryptococcal Ag, India ink, HSV PCR, and TB. Laboratory testing by Mayo Clinic revealed that the serum anti-NMDA antibodies were negative, but CSF anti-NMDA antibodies with repeat testing were found to be positive. Treatment included bilateral salpingo-oophorectomy (but no ovarian teratoma was found), IVIG transfusion, and plasmapheresis. She was later switched to rituximab infusion and also received medications for psychosis and seizures. Final diagnosis was documented as anti-NMDA-receptor encephalitis without teratoma. A neuropsychological evaluation occurred after treatment. It showed numerous cognitive deficits such as impairments in visual memory, mental flexibility, expressive language, visuospatial skills, and motor dexterity (see Table 1). The most significant problems were present in verbal fluency (semantic and phonemic fluency, both <1st percentile), mental flexibility (<1st percentile), and elevated depression and anxiety. She continues to have compromised cognitive status along with residual frontal deficits and mood changes. She and her guardian provided consent for this case study to be published.

## 3. Discussion

In anti-NMDAR encephalitis, antibodies are directed towards the NR1 subunit of the NMDA receptor causing a depletion of NMDA receptors [1, 4, 6, 7]. The lack of receptors causes neuropsychiatric symptoms, attributable to ovarian teratomas and autoimmune processes in approximately half of the cases [2–4, 6, 8]. Recognizing the symptom complex is crucial to diagnosis [7]. Commonly, patients develop a multistage illness that progresses from psychiatric symptoms to more extreme global symptoms [1]. Up to half of the women diagnosed with anti-NMDAR encephalitis have an underlying ovarian teratoma [3, 8].

Treatment involves immunosuppression with steroids and intravenous administration [1, 4, 7, 9]. Many patients recover well after appropriate treatment [10]. While it is thought that there is a higher prevalence of anti-NMDAR encephalitis, data on posttreatment cognitive states remain rather limited [11, 12]. This case revealed residual frontal deficits with mood changes in the context of anti-NMDAR encephalitis. This case is the first to describe posttreatment cognition in anti-NMDAR encephalitis after probable serial autoimmune episodes.

## 4. Conclusion

Anti-N-methyl-D-aspartate receptor encephalitis is a neurological, autoimmune disorder tightly conceptualized only as recently as the mid-2000s. It presents itself in a combination of psychiatric, neurological, and autonomic features. This case was a unique case with probable earlier episode (prior to the mid-2000s operationalization of the disease) and a later relapse, accompanying a comprehensive neuropsychological profile tracked after the relapse and subsequent improvement. Neurocognitive findings revealed residual frontal deficits with mood changes even in the state after plasmapheresis, and these deficits do not appear to associate neatly with structural brain damage visible in imaging (or lack thereof), as clinical symptoms were likely the sequelae of a more global neuroinflammatory damage that occurred. This case is the first to describe posttreatment cognition in anti-NMDAR encephalitis after probable serial autoimmune episodes.

## Figures and Tables

**Table 1:** Neuropsychological test scores.

Domain	Function	Measure^*∗*^	Standard scores	Percentile
Expected intelligence	Estimated premorbid IQ	WRAT-4 Reading subtest	86	18

General intelligence	Full scale IQ	WAIS- IV	75	5

Verbal memory	Total verbal learning	CVLT-II trials 1–5 total	98	45
	New learning as distraction to old learning	CVLT-II trial B	107	68
	Short delay recall	CVLT-II short delay	78	7
	Long delay recall	CVLT-II long delay	85	16
	Recognition	CVLT-II discriminability	115	84

Visual memory	Total visual learning	BVMT-R trials 1–3 total	<65	<1
	Delay recall	BVMT-R delay	<65	<1
	Recognition	BVMT-R Discrimination Index	—	1-2

Attention	Working memory	WAIS-IV Working Memory Index	80	9
	Psychomotor speed & attention	Trails A	88	21

Higher order executive skills	Mental flexibility	Trails B	58	.3
	Response inhibition	Stroop C-W	91	27
	Novel problem solving	WCST total error	78	7
		WCST-64 perseverative responses	86	18
		WCST-64 perseverative errors	87	9
		WCST-64 categories completed	—	2–5
		WCST-64 failure to maintain set	—	—

Speed	Processing speed	WAIS-IV Processing Speed Index	86	18
	Recognition speed	Stroop W	84	14
		Stroop C	86	16

Verbal general	Verbal comprehension	WAIS-IV Verbal Comprehension Index	83	13

Expressive	Semantic fluency	Animals	58	.3
	Phonemic fluency	FAS	58	.3
	Naming	BNT-2	64	.8
	Repetition	MAE sentences	71	3

Receptive	Comprehension	MAE token	106	67

General perception	Perceptual reasoning	WAIS-IV Perceptual Reasoning Index	71	3

Visuospatial construction	Visuomotor construction	Clock drawing	—	—

Spatial perception	Visual perception	JOLO	79	8

Motor	Manual dexterity	GD dominant hand	67	1.4
		GD nondominant hand	64	.8

^*∗*^WRAT-4 Reading = Wide Range Achievement Test, 4th Edition; WAIS-IV = Wechsler Adult Intelligence Scale, 4th Edition; CVLT-II = California Verbal Learning Test, 2nd Edition; BVMT-R = Brief Visuospatial Memory Test-Revised; Trails A = Trail Making Test Form A; Trails B = Trail Making Test Form B; Stroop C = Stroop Color Subtest; Stroop W = Stroop Word Subtest; Stroop C-W = Stroop Color-Word Subtest; WCST = Wisconsin Card Sorting Test; Animals = semantic fluency; FAS = phonemic fluency; BNT-2 = Boston Naming Test, 2nd Edition; MAE = Multilingual Aphasia Examination; JOLO = Judgment of Line Orientation Test; GD = grip dynamometer.
